# Supplementary material for: Structural basis for kinase inhibition in the tripartite E. coli HipBST toxin–antitoxin system
Source: eLife. 2023 Nov 6;12:RP90400. doi: 10.7554/eLife.90400 (PMC10627512; doi:10.7554/eLife.90400)
Supplement: Figure 1—figure supplement 2—source data 1. [file elife-90400-fig1-figsupp2-data1.zip › Figure 1-figure supplement 2-source data 1/Figure 1-figure supplement 2-source data 1.pptx]

## Slide 1
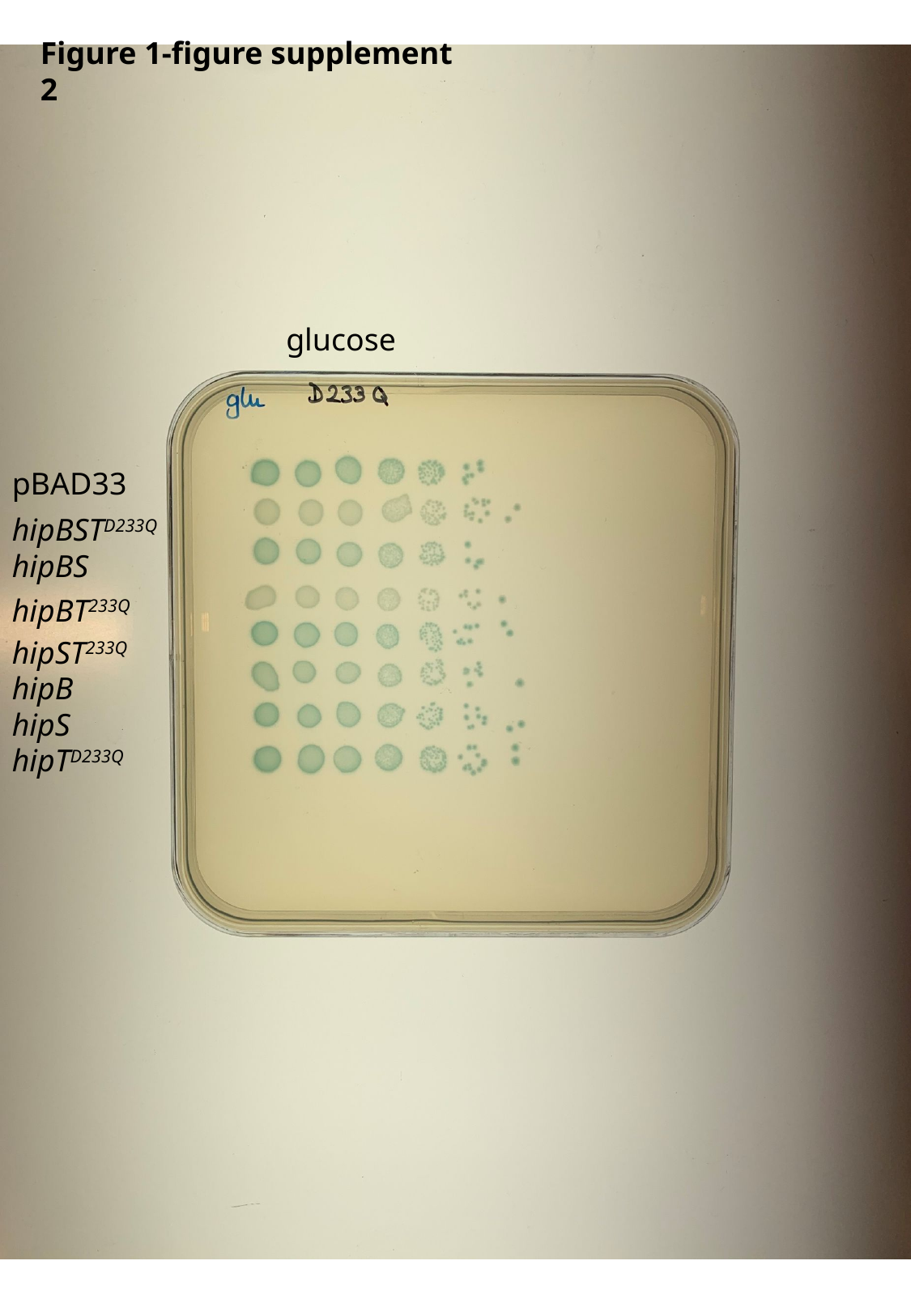

Figure 1-figure supplement 2
glucose
pBAD33
hipBSTD233Q
hipBS
hipBT233Q
hipST233Q
hipB
hipS
hipTD233Q

## Slide 2
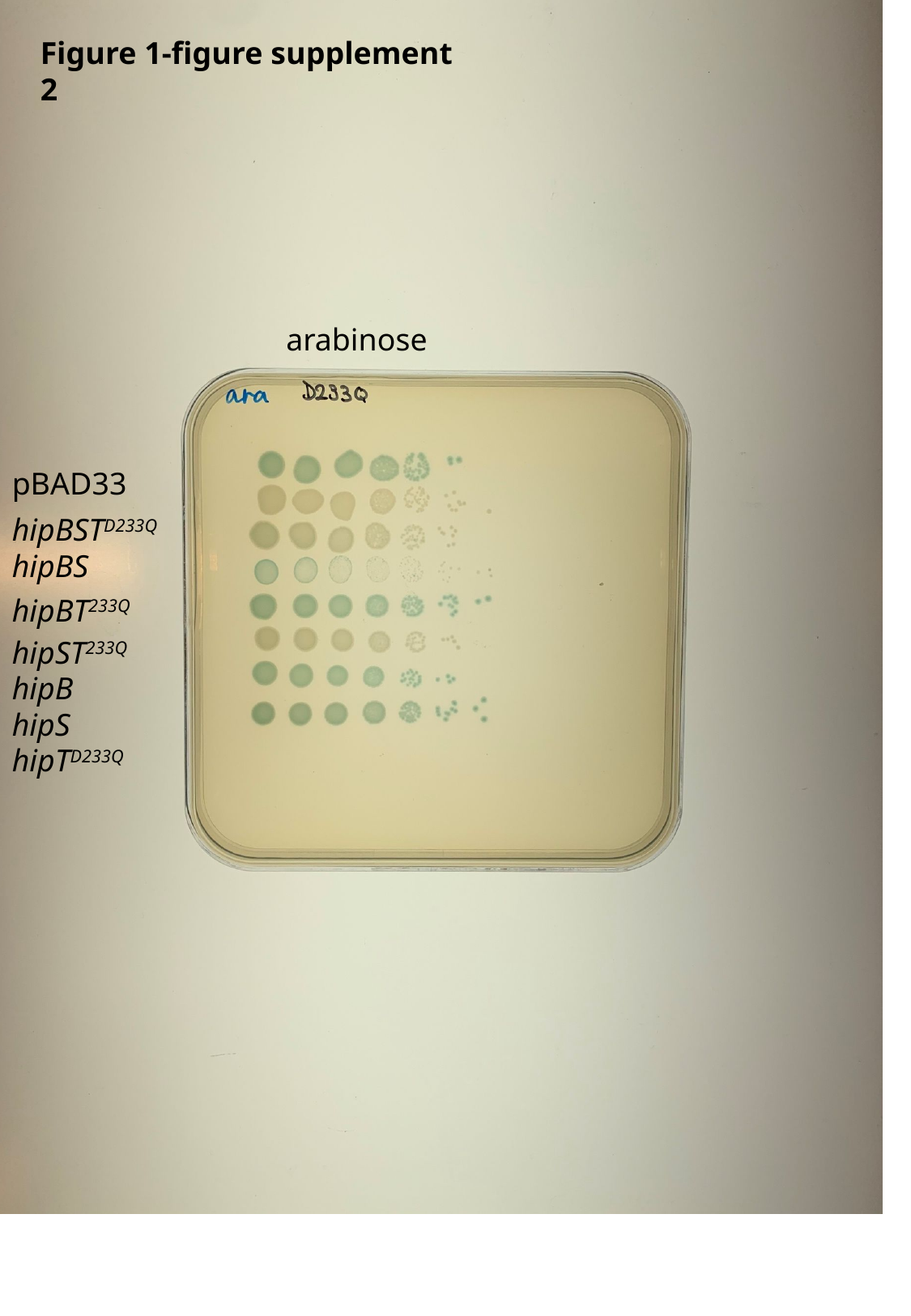

Figure 1-figure supplement 2
arabinose
pBAD33
hipBSTD233Q
hipBS
hipBT233Q
hipST233Q
hipB
hipS
hipTD233Q
