# Supplementary figures and images for: Structural basis for kinase inhibition in the tripartite E. coli HipBST toxin–antitoxin system
Source: eLife. 2023 Nov 6;12:RP90400. doi: 10.7554/eLife.90400 (PMC10627512; doi:10.7554/eLife.90400)

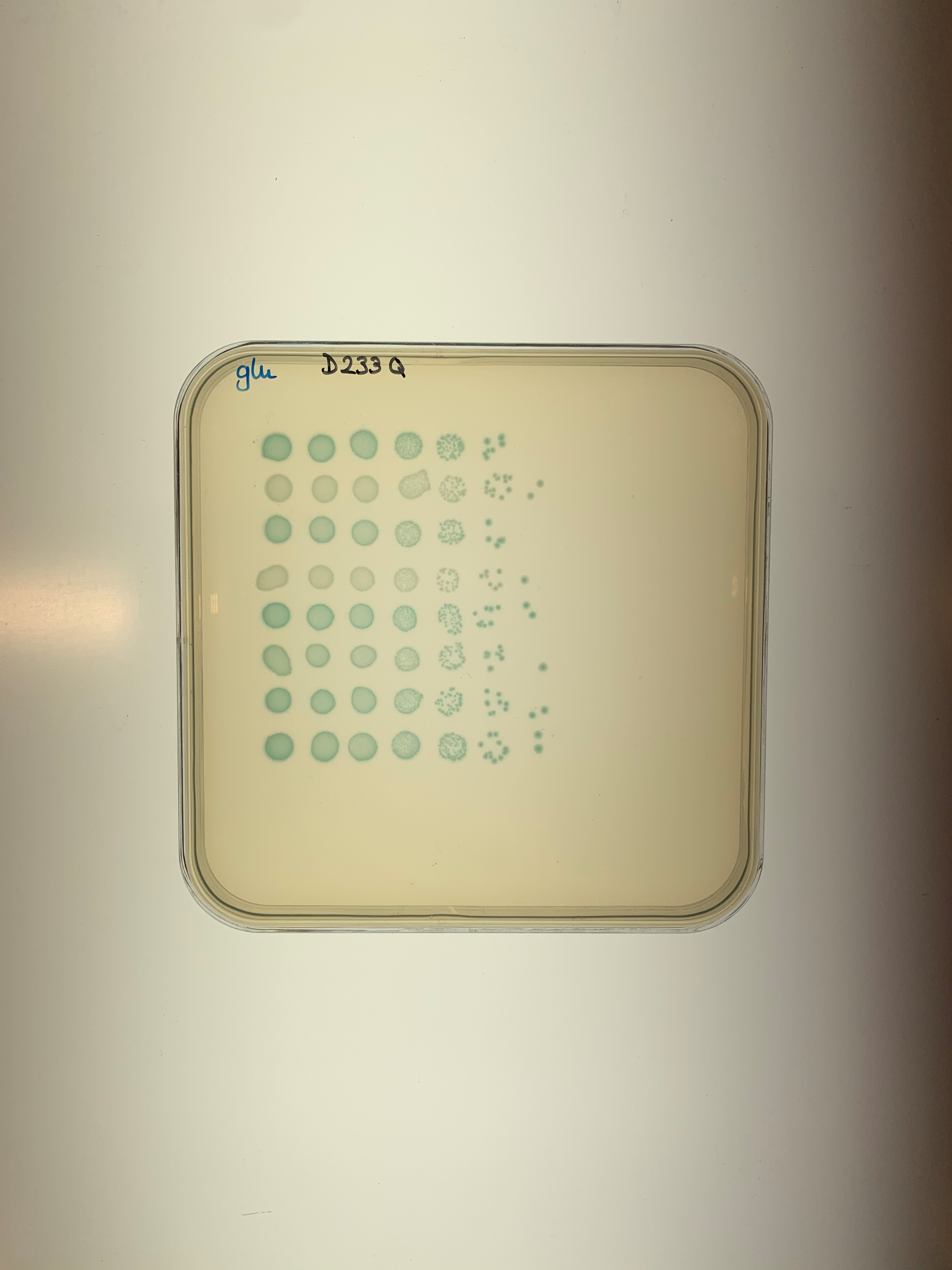

Supplement: Figure 1—figure supplement 2—source data 1. [file elife-90400-fig1-figsupp2-data1.zip › Figure 1-figure supplement 2-source data 1/Figure 1-figure supplement 2-source data 1-1.tif]

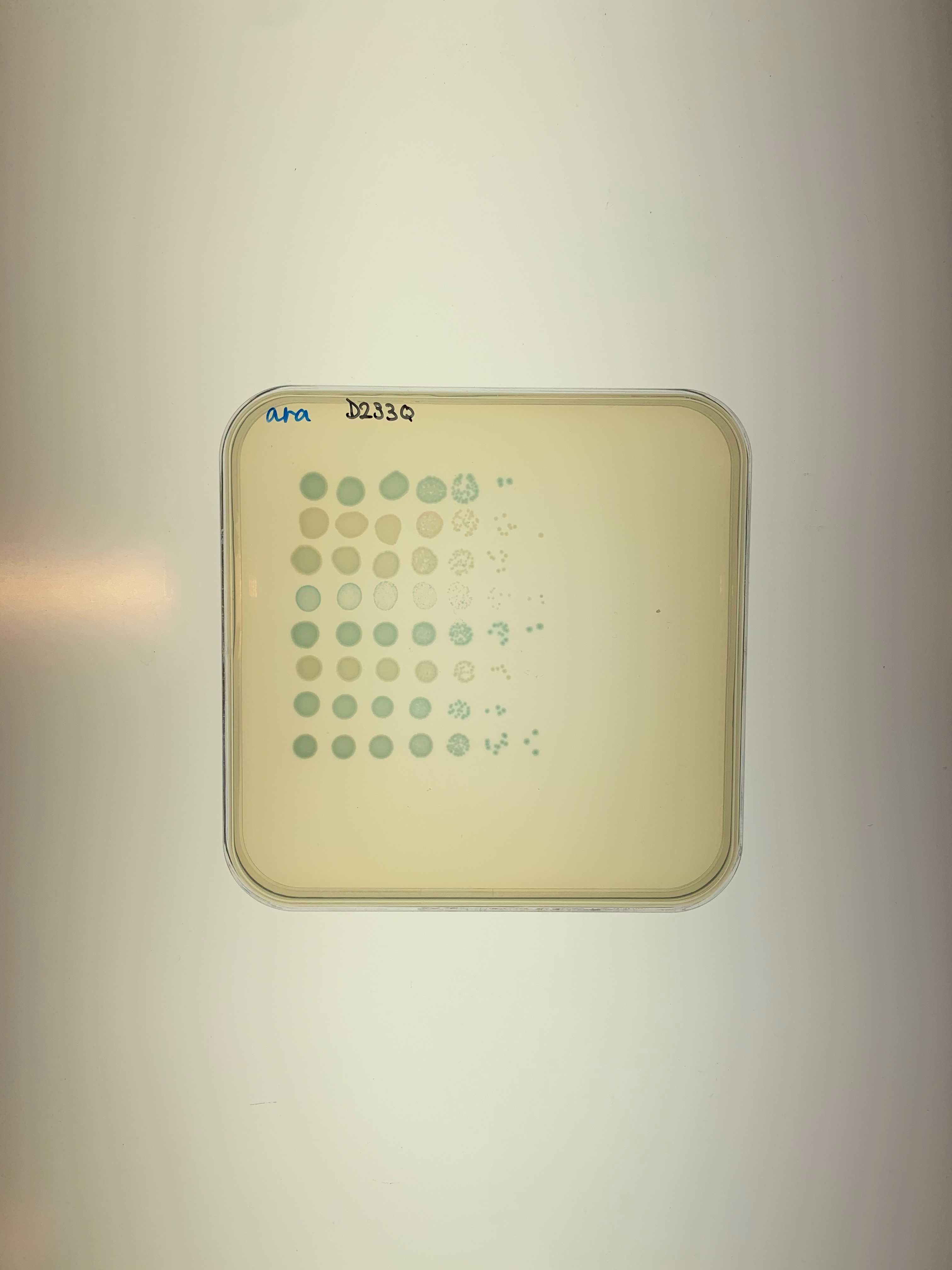

Supplement: Figure 1—figure supplement 2—source data 1. [file elife-90400-fig1-figsupp2-data1.zip › Figure 1-figure supplement 2-source data 1/Figure 1-figure supplement 2-source data 1-2.tif]

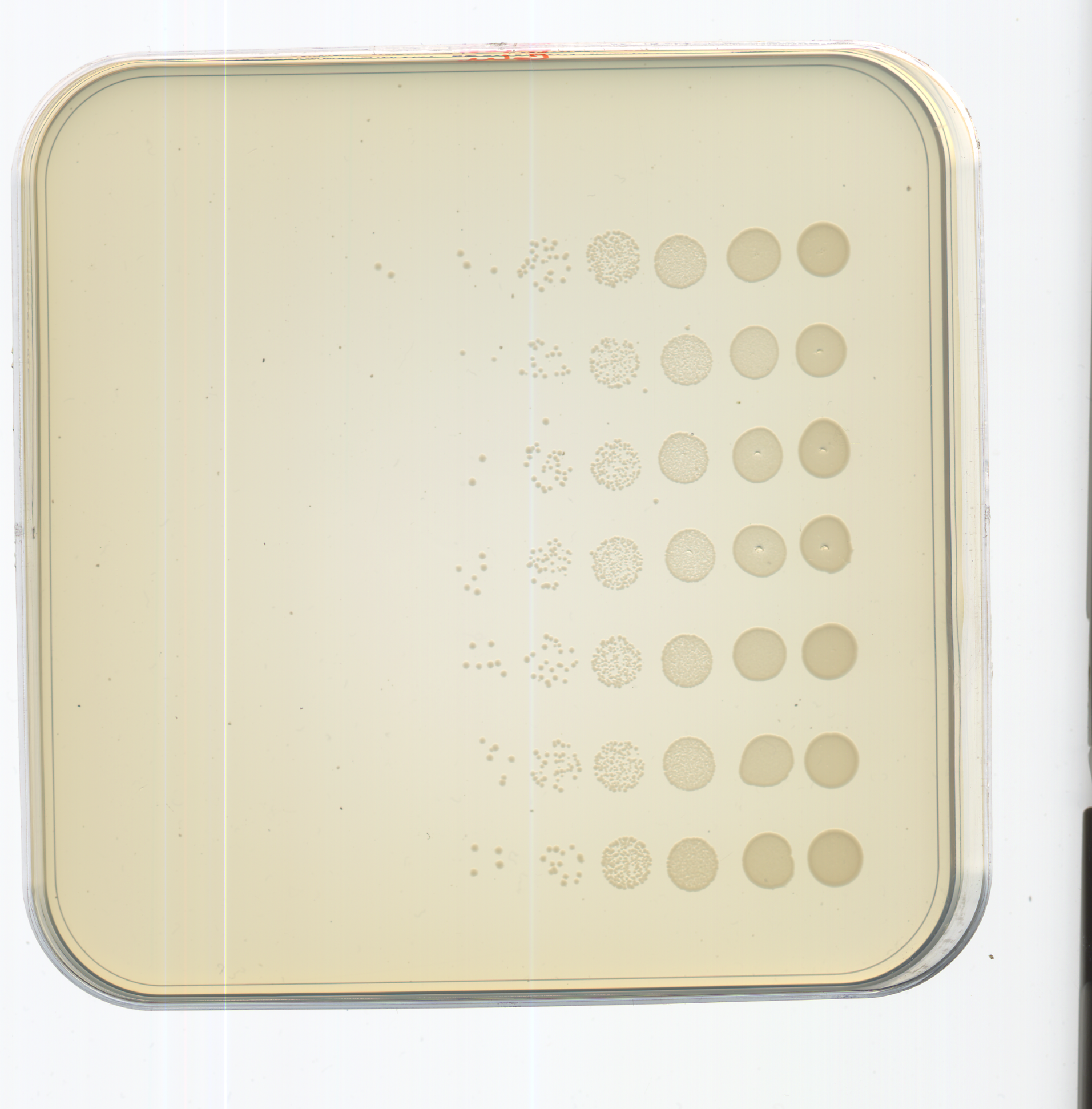

Supplement: Figure 2—source data 1. [file elife-90400-fig2-data1.zip › Figure 2-source data 1/Figure 2-source data 1-1.tif]

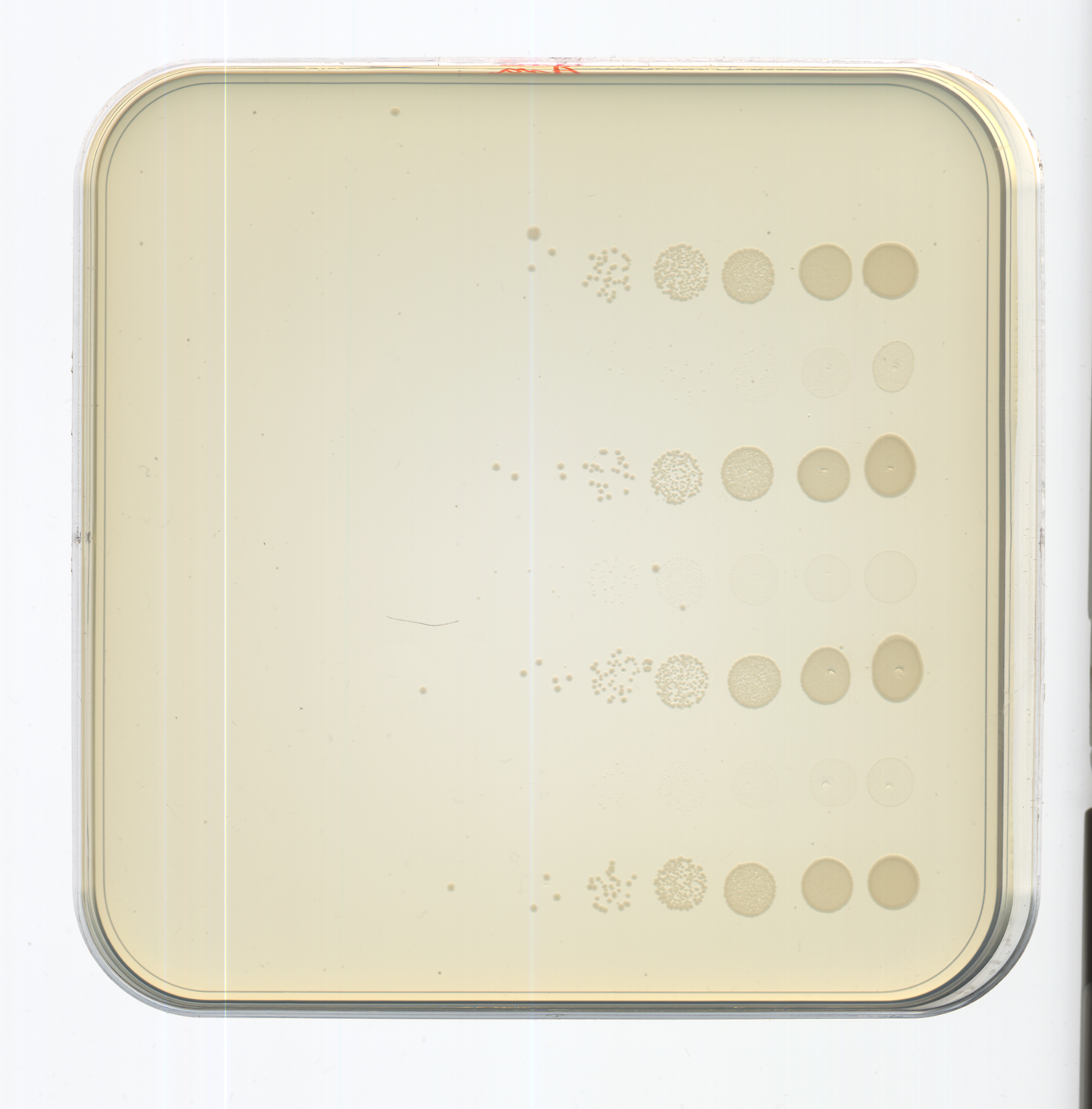

Supplement: Figure 2—source data 1. [file elife-90400-fig2-data1.zip › Figure 2-source data 1/Figure 2-source data 1-2.tif]

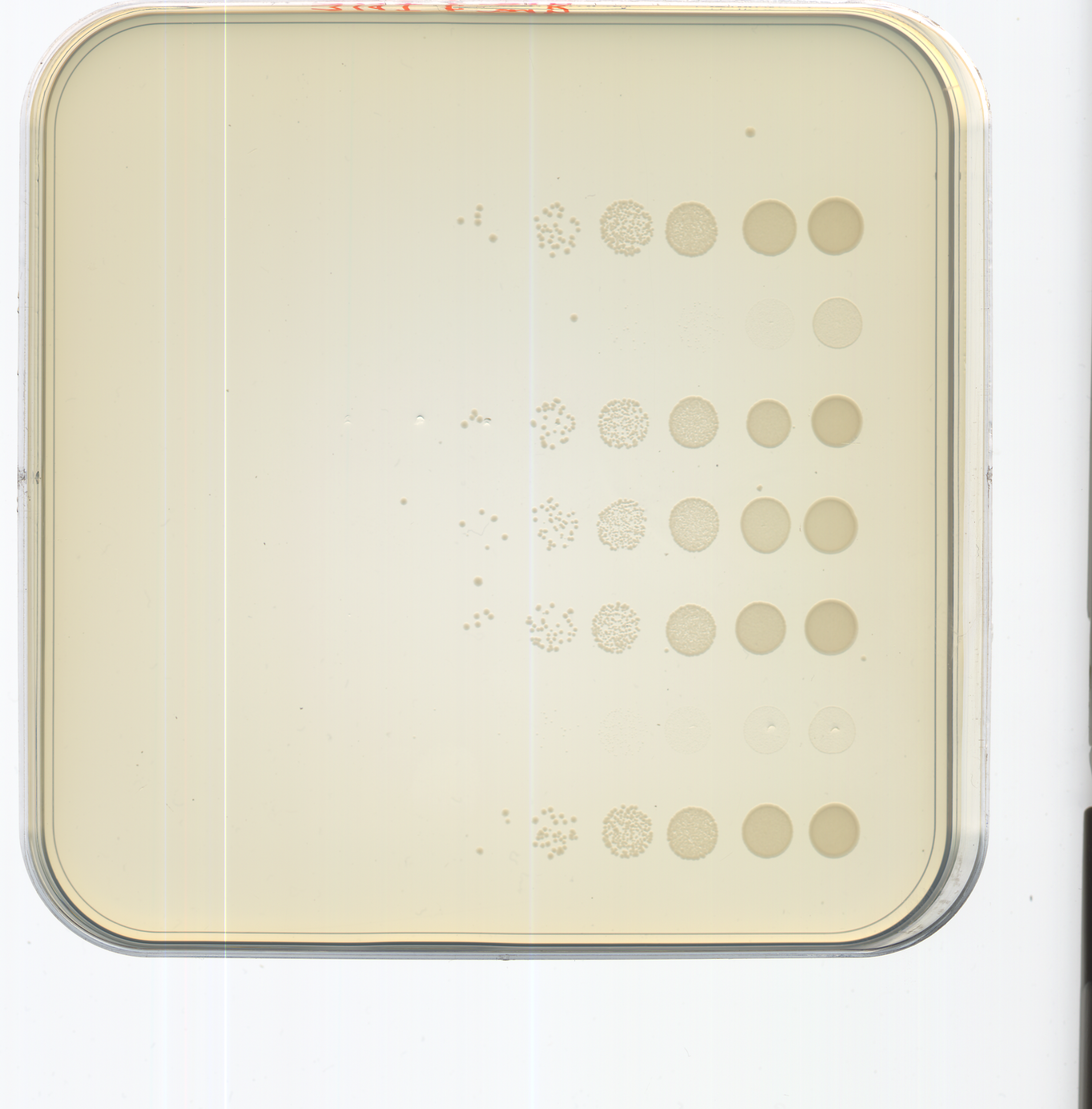

Supplement: Figure 2—source data 1. [file elife-90400-fig2-data1.zip › Figure 2-source data 1/Figure 2-source data 1-3.tif]

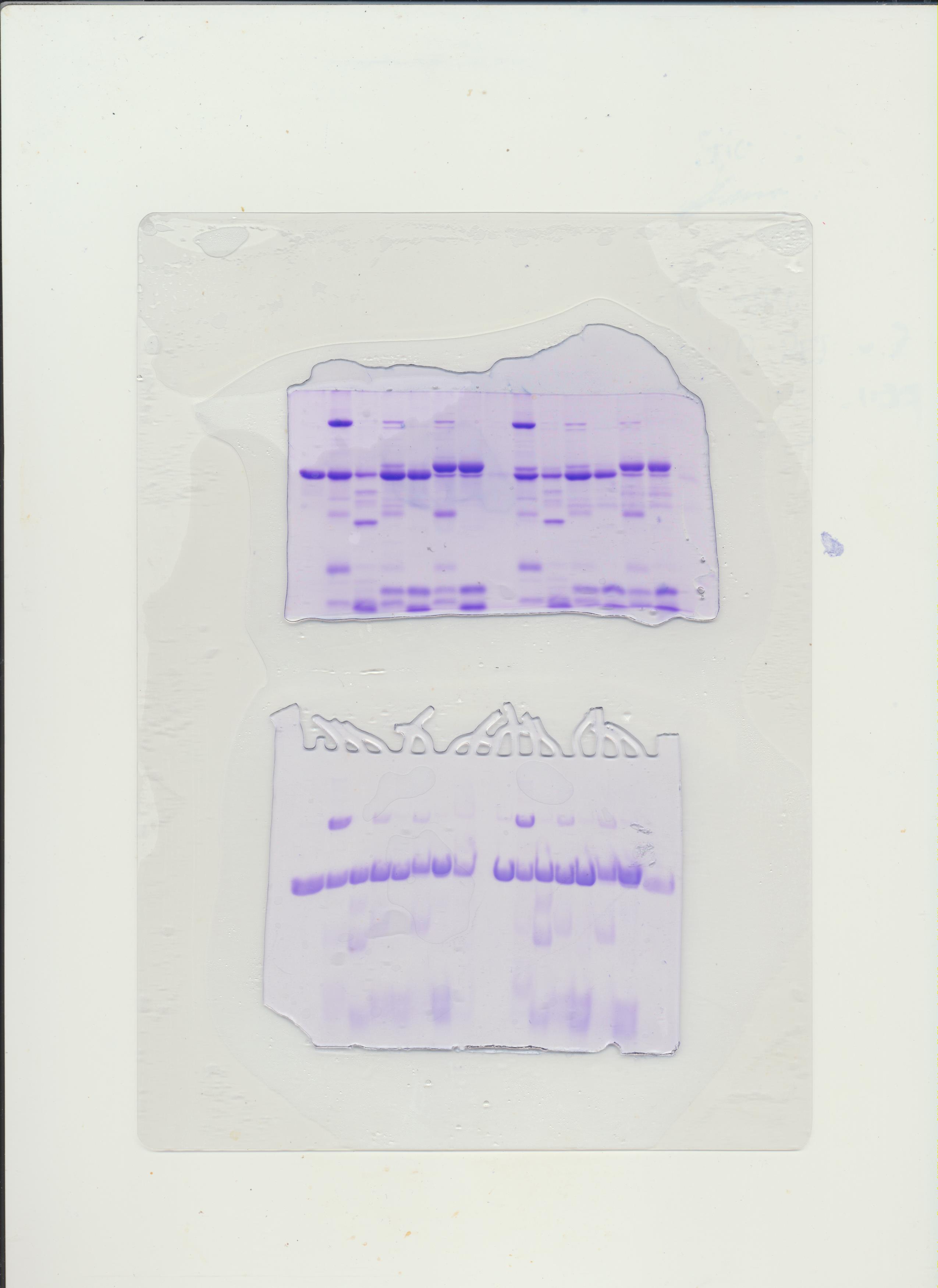

Supplement: Figure 3—source data 1. [file elife-90400-fig3-data1.zip › Figure 3-source data 1/Figure 3-source data 1.jpg]

## Slide 1
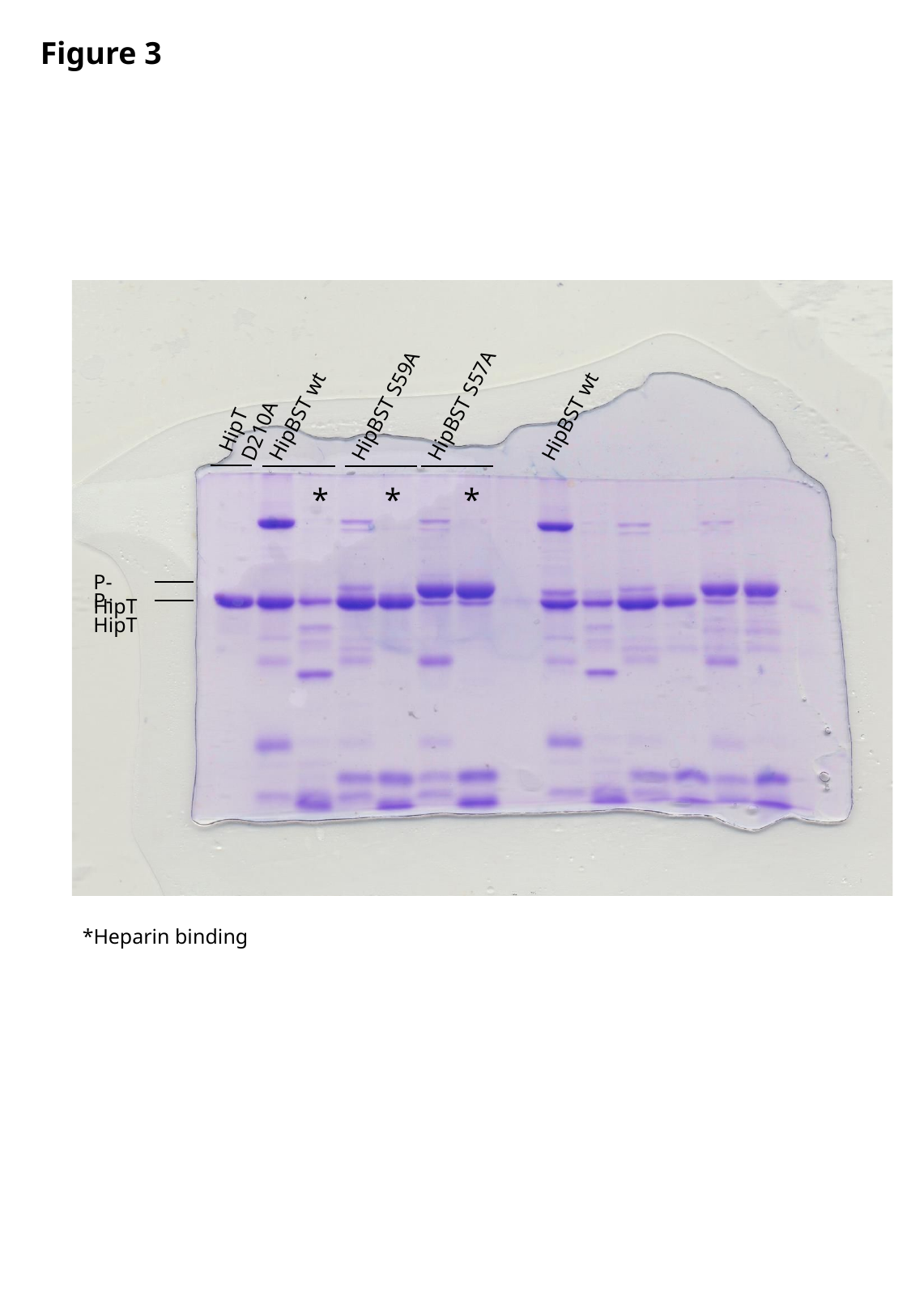

Figure 3
HipBST S59A
HipT D210A
HipBST S57A
HipBST wt
HipBST wt
*
*
*
P-HipT
P-HipT
*Heparin binding

Supplement: Figure 3—source data 1. [file elife-90400-fig3-data1.zip › Figure 3-source data 1/Figure 3-source data 1.pptx]

## Slide 1
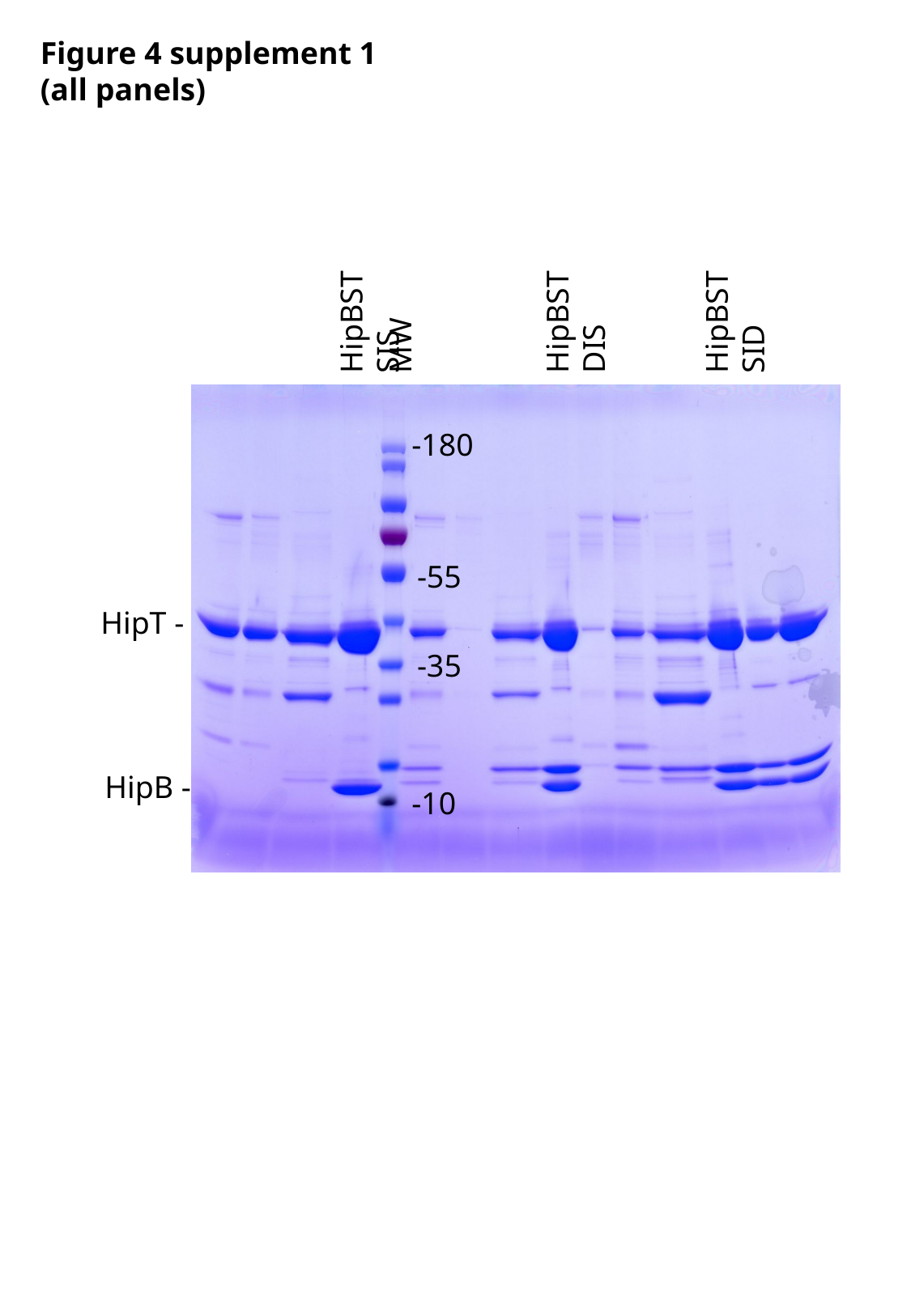

Figure 4 supplement 1
(all panels)
HipBST DIS
HipBST SID
HipBST SIS
MW
-180
-55
HipT -
-35
HipB -
-10

Supplement: Figure 4—figure supplement 1—source data 1. [file elife-90400-fig4-figsupp1-data1.zip › Figure 4-figure supplement 1-source data 1/Figure 4-figure supplement 1-source data 1.pptx]

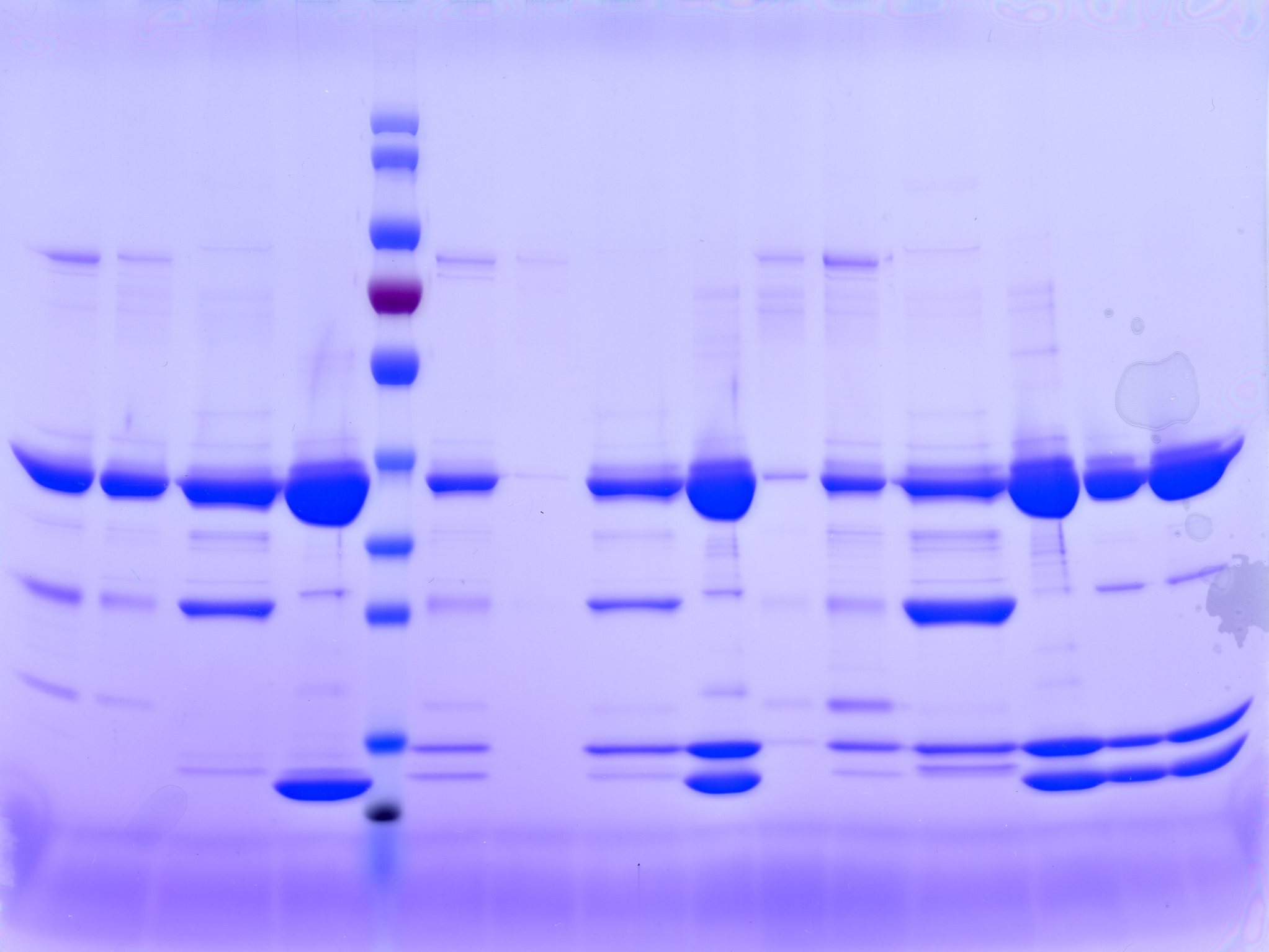

Supplement: Figure 4—figure supplement 1—source data 1. [file elife-90400-fig4-figsupp1-data1.zip › Figure 4-figure supplement 1-source data 1/Figure 4-figure supplement 1-source data 1.jpg]
