# Supplementary material for: Structural basis for kinase inhibition in the tripartite E. coli HipBST toxin–antitoxin system
Source: eLife. 2023 Nov 6;12:RP90400. doi: 10.7554/eLife.90400 (PMC10627512; doi:10.7554/eLife.90400)
Supplement: Figure 2—source data 1. [file elife-90400-fig2-data1.zip › Figure 2-source data 1/Figure 2-source data 1.pptx]

## Slide 1
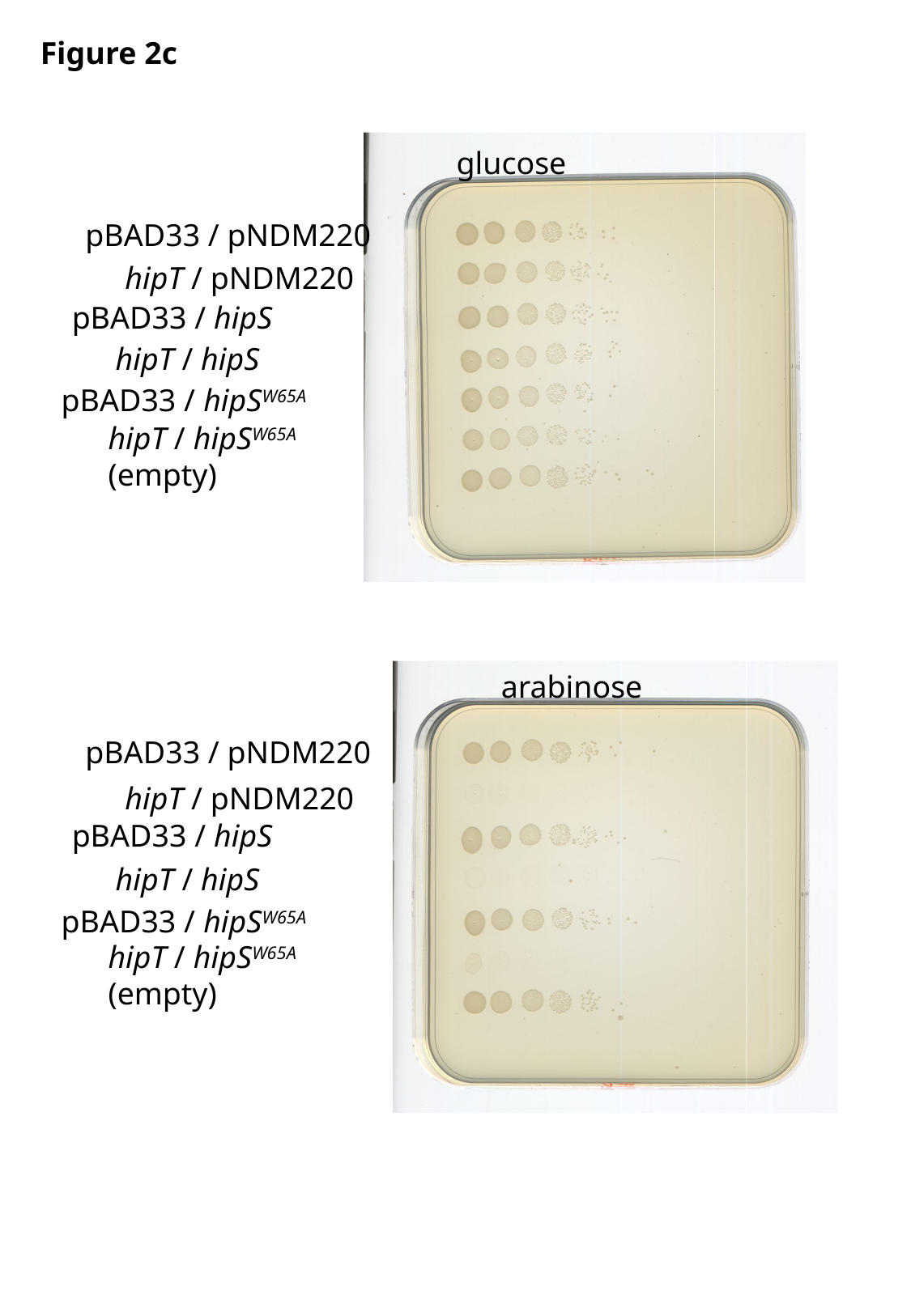

Figure 2c
glucose
pBAD33 / pNDM220
hipT / pNDM220
pBAD33 / hipS
hipT / hipS
pBAD33 / hipSW65A
hipT / hipSW65A
(empty)
arabinose
pBAD33 / pNDM220
hipT / pNDM220
pBAD33 / hipS
hipT / hipS
pBAD33 / hipSW65A
hipT / hipSW65A
(empty)

## Slide 2
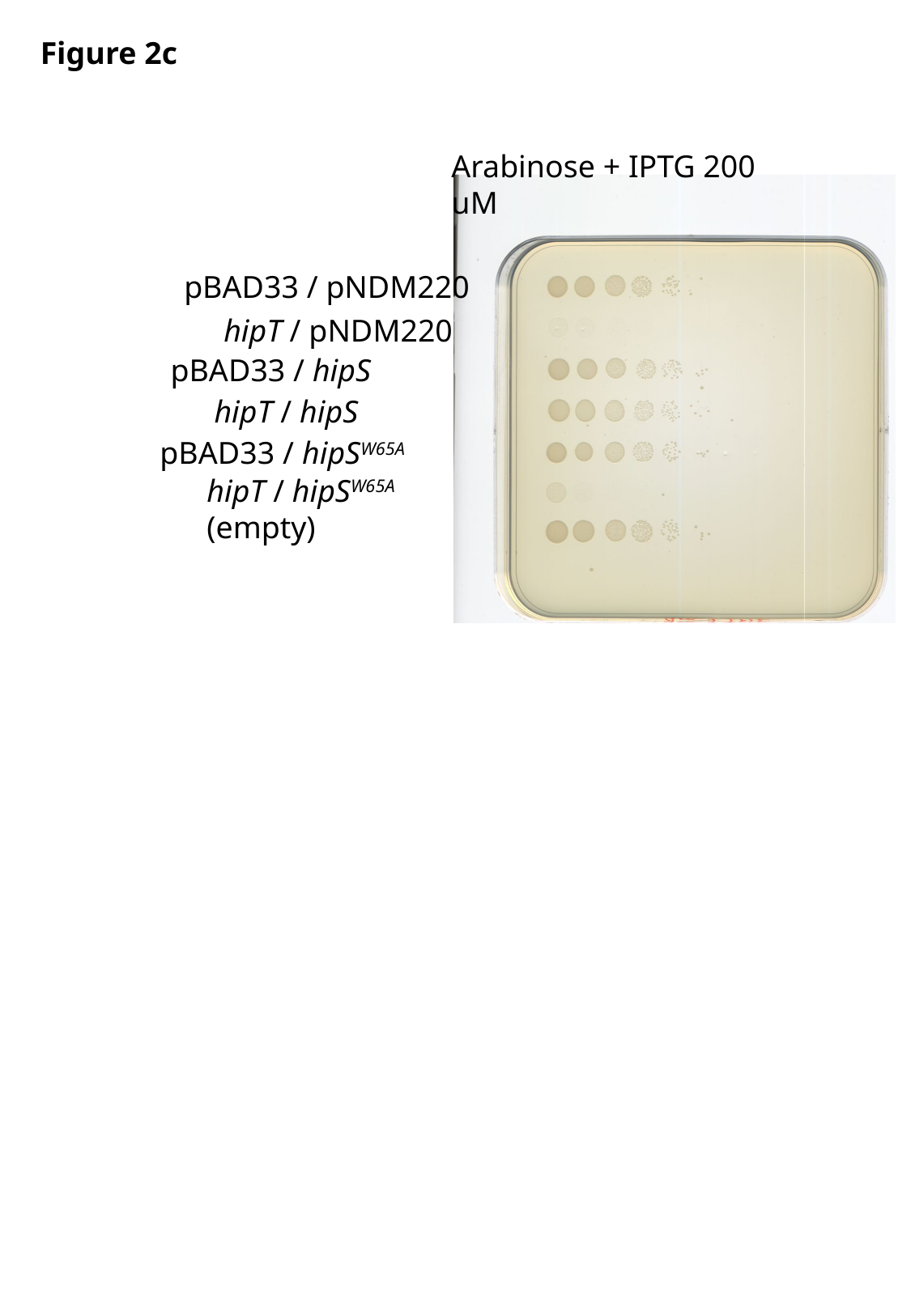

Figure 2c
Arabinose + IPTG 200 uM
pBAD33 / pNDM220
hipT / pNDM220
pBAD33 / hipS
hipT / hipS
pBAD33 / hipSW65A
hipT / hipSW65A
(empty)
