# Supplementary material for: Structural basis for kinase inhibition in the tripartite E. coli HipBST toxin–antitoxin system
Source: eLife. 2023 Nov 6;12:RP90400. doi: 10.7554/eLife.90400 (PMC10627512; doi:10.7554/eLife.90400)
Supplement: Supplementary file 1. — (a) Crystallographic data statistics. Crystallographic data collection (upper part) and refinement (lower part) statistics for the HipBSTD233Q, HipBSTS57A, and HipBSTS59A structures. *Numbers in parentheses refer to the outermost resolution shell. (b) Bacterial strains and plasmids. List of bacterial strains and plasmids either prepared as part of this work or with the given reference. SD, Shine-Dalgarno sequence. (c) Oligonucleotides and primers. List of oligonucleotides and primers used in this work, 5′–3′ sequences. [file elife-90400-supp1.docx]

**Supplementary file 1a**

|  | **HipBST^D233Q^** | **HipBST^S57A^** | **HipBST^S59A^** |
| --- | --- | --- | --- |
| ***Data Collection***  Wavelength (Å)  Resolution range (Å)  Space group  Unit cell dimensions  a, b, c, (Å)  *α, β, γ* (^o^) | 1.54180  49.9–2.9 (3.0–2.9)*  C121  281.66,106.47, 57.75 90, 90.75, 90 | 0.97625  46.9–2.4 (2.5–2.4)*  C121  281.68, 106.07, 57.56 90, 90.65, 90 | 1.54180  50.4–3.34 (3.5 – 3.4)*  C121  285.26, 107.15, 58.45  90, 90.71, 90 |
| Total reflections | 74,587 (7,335) | 625,868 (25,597) | 41,777 (4,068) |
| Unique reflections | 37,716 (3,719) | 66,117 (6,503) | 22,580 (2,193) |
| Multiplicity | 2.0 (2.0) | 9.5 (3.9) | 1.9 (1.9) |
| Completeness (%) | 99.8 (99.3) | 99.9 (99.4) | 87.71 (84.84) |
| R_meas_ (%) | 0.13 (1.105) | 0.27 (0.08) | 0.17 (1.01) |
| I/σ(I) | 6.62 (0.86) | 10.3 (0.72) | 7.10 (0.93) |
| CC_1/2_ | 0.99 (0.37) | 0.99 (0.37) | 0.97 (0.43) |
| ***Refinement***  Average B-factor (Å^2^)  protein  ligands  solvent  No. of reflections  No. of reflections (free)  R-work (%)  R-free (%)  Number of  protein (residues)  solvent (atoms)  ligand (atoms)  rmsd (bonds, Å)  rmsd (angles, degrees)  Rotamer outliers (%)  Clashscore  Ramachandran statistics  favoured (%)  allowed (%)  outliers (%)  Rama-Z score  whole  helix  sheet  loop | 98.5  98.9  -  80.0  37,706 (3,719)  1,914 (175)  19.5 (31.8)  22.8 (34.5)  1,018  203  -  0.011  1.49  9.8  4.4  95.1  4.7  0.2  -1.63  -1.27  -1.36  -0.75 | 86.1  86.5  166.7  76.7  66,112 (6,504)  3,364 (326)  21.0 (42.8)  23.8 (46.2)  1,017  402  20 0.013  1.75 6.0  2.7  96.0  3.6  0.4  -0.44  -0.40  0.21  -0.16 | 119.4  119.9  118.6  78.3  22,570 (2,193)  1086 (86)  20.2 (29.2)  24.3 (35.4)  1,023  110  10  0.013  1.66  10.07  5.8  95.6  3.9  0.5  -0.90  -0.48  0.07  -0.73 |

**Supplementary file 1b**

| *E. coli* strains | Description | Reference or source |
| --- | --- | --- |
| MG1655 | Wild-type K12 | (Blattner *et al.*, 1997) |
| TB28 | MG1655 *∆lacIZYA* | Laboratory collection |

| Plasmid | Description | Reference or source |
| --- | --- | --- |
| pBAD33 | p15 *araC* P_BAD_, Cm^r^ | (Guzman *et al.*, 1995) |
| pGH254 | Mini-R1, *lacZYA* transcriptional fusion vector, Kan^r^ | Laboratory collection |
| pNDM220 | Mini-R1 *lacI*^q^ P_A1/04/03_, Amp^r^ | (Gotfredsen & Gerdes, 1998) |
| pET-15b | pBR322 *lacI* P_T7_, Amp^r^ | Novagen |
| pKG127 | pUC57::*hipBST_O127_* | (Vang Nielsen *et al.*, 2019) |
| pSVN1 | pBAD33::*hipT*, start codon GTG | (Vang Nielsen *et al.*, 2019) |
| pSVN68 | pUC57::*hipB*-*S*-*T*^S57A^_His6_, optimized SDs for all genes | This work |
| pSVN78 | pET-15b::*hipB*-*S*-*T*^S57A^_His6_, optimized SDs for all genes | This work |
| pSVN88 | pUC57::*hipB*-*S*-*T*^D233Q^_His6_, optimized SDs for all genes | This work |
| pSVN96 | pET-15b::*hipB*-*S*-*T*^D233Q^_His6_, optimized SDs for all genes | This work |
| pSVN109 | pNDM220::*hipS*, optimized SD | (Vang Nielsen *et al.*, 2019) |
| PSVN141 | pGH254::P*_hipBST_*-*hipB*’-*laZ*, transcriptional P*_hipBST_*-*hipB*’ *lacZ* fusion | This work |
| pSVN178 | pNDM220::*hipS*^W65A^, optimized SD | This work |
| pSVN181 | pBAD33::*hipB*-*S*-*T*^D233Q^, optimized SDs for all genes | This work |
| pSVN182 | pBAD33::*hipB*-*S*, optimized SDs for both genes | This work |
| pSVN185 | pBAD33::*hipB*-*T*^D233Q^, optimized SDs for both genes | This work |
| pSVN188 | pBAD33::*hipS*-*T*^D233Q^, optimized SDs for both genes | This work |
| pSVN189 | pBAD33::*hipB*, optimized SD | This work |
| pSVN190 | pBAD33::*hipS*, optimized SD | This work |
| pSVN193 | pBAD33::*hipT*^D233Q^, optimized SD | This work |
| pSVN194 | pBAD33::*hipT*^S57D^, start codon GTG | This work |
| pSVN195 | pBAD33::*hipT*^S59D^, start codon GTG | This work |
| pSVN199 | pBAD33::*hipT*^S57A^, start codon GTG | This work |
| pSVN201 | pBAD33::*hipT*^S59A^, start codon GTG | This work |
| pSNN1 | pET-15b::*hipT*^S57A^_His6_, optimized SD | This work |
| pSNN2 | pET-15b::*hipT*^S57A+D210A^_His6_, optimized SD | This work |
| pMME3 | pET-15b::*hipB-S-T*^S59A^_His6_, optimized SDs for all genes | This work |
| pRBS1 | pET-15b::*hipB*-*S*-*T*^D210A^_His6_, optimized SDs for all genes | This work |
| pRBS2 | pET-15b::*hipB*-*S*-*T*^S57D,D210A^_His6_, optimized SDs for all genes | This work |
| pRBS3 | pET-15b::*hipB*-*S*-*T*^S59D,D210A^_His6_, optimized SDs for all genes | This work |

**Supplementary file 1c**

| Oligonucleotide | Sequence |
| --- | --- |
| FP1(GTG) | CCCCGTCGACGGATCCAAGGAGTTTTATAAGTGGCGAATTGTCGTATTCTG |
| FP21 | GGGGGGTACCGGATCCAAAATAAGGAGGAAAAAAAAATGATCTGCTCAGGACCAC |
| FP22 | CCCCCTCGAGGGATCCAAAATAAGGAGGAAAAAAAAATGCATCGGCGAGTGAAAG |
| FP43 | CCCCGAATTCCTCTCCCGATGAGATCAGC |
| FP46 | GGGGGTCGACCTGCAGAAAATAAGGAGGAAAAAAAAATGGCGAATTGTCGTATTCTG |
| FP47 | GGGGGGTACCGGATCCAAAATAAGGAGGAAAAAAAAATGGCGAATTGTCGTATTCTG |
| FP48 | GGGGGTCGACCTGCAGAAAATAAGGAGGAAAAAAAAATGGCGAATTGTCGTATTCTG |
| RP1 | CCCCCGCATGCGAATTCGCTCACAGCAGCCCCAGACG |
| RP11 | CCCCCTCGAGAAGCTTTCACAGCAGCCCCAGACG |
| RP14 | GGGGGAATTCAAGCTTTTATTCCTCCCAAGGTAAAATC |
| RP15 | GGGGGAATTCAAGCTTTCACTCGCCGATGCATAG |
| RP32 | CCCCGGATCCTCTGCAACTCCTGGAGTTG |
| RP42 | GGGGGTCGACCTGCAGTCACTCGCCGATGCATAG |
| HipT S57D Fw | GCGTCAACAAAAAGGGATGGATATTTCCGGTT |
| HipT S57D Rv | GGGCTGGTAACCGGAAATATCCATCCCTTTTT |
| HipT S59D Fw | GTCAACAAAAAGGGATGAGTATTGACGGTTAC |
| HipT S59D Rv | TTGGGCTGGTAACCGTCAATACTCATCCCTTT |
| HipT S59A Fw | GTCAACAAAAAGGGATGAGTATTGCCGGTTAC |
| HipT S59A Rv | TTGGGCTGGTAACCGGCAATACTCATCCCTTT |
| hipT D210A Fw | TAAATGCATCGCGTTATTACCCAGCAACAA |
| hipT D210A Rv | CTGGGTAATAACGCGATGCATTTACGAAACTTT |
| hipT S57S59A Fw | GGGATGAGTATTGCCGGTTACCAGCCCAAATTGCAA |
| hipT S57S59A Rv | GTAACCGGCAATACTCATCCCTTTTTGTTGACGCGG |
| hipS W65A Fw | CAGAAGGAGCTCTGCGTCAACGCTA |
| hipS W65A Rv | TGACGCAGAGCTCCTTCTGGCGC |
| hipX S57A Fw | AAGGGATGGCTATTTCCGGTTACCAGCC |
| hipX S57A Rv | CGGAAATAGCCATCCTTTTTGTTGACG |
| hipX D233Q Fw | CGGTGTATCAGTTTGTTTCTGTCGCTCCC |
| hipX D233Q Rv | GAAACAAACTGATACACCGGCGCTAACG |
| hipBS del Fw | ACGACAATTCGCCATTTTTTTTTCCTCCTTATTTTTCTAGAGGG |
| hipBS del Rv | TTCCCCTCTAGAAAAATAAGGAGGAAAAAAAAATGGCGAAT |
| Q5 HipT D210A Fw | GGTAATAACGctATGCATTTACGAAACTTTG |
| Q5 HipT D210A Rv | CAGCAACCAGGCGTAAAC |
| Q5 HipT S57D Fw | AAAGGGATGGaTATTTCCGGT |
| Q5 HipT S57D Rv | TTGTTGACGCGGAAGTTC |
| Q5 HipT S59D Fw | tagaCATCCCTTTTTGTTGACG |
| Q5 HipT S59D Rv | ttgatGGTTACCAGCCCAAATTG |
